# Consolidated criteria for strengthening reporting of health research involving indigenous peoples: the CONSIDER statement

**DOI:** 10.1186/s12874-019-0815-8

**Published:** 2019-08-09

**Authors:** Tania Huria, Suetonia C. Palmer, Suzanne Pitama, Lutz Beckert, Cameron Lacey, Shaun Ewen, Linda Tuhiwai Smith

**Affiliations:** 1 Māori and Indigenous Health Institute, University of Otago Christchurch, 2 Riccarton Ave, Christchurch, 8140 New Zealand; 2 Department of Medicine, University of Otago Christchurch, Christchurch, New Zealand; 3 Melbourne Poche Centre for Indigenous Health, The University of Melbourne, Melbourne, Australia; 4 Te Kotahi Research Institute, University of Waikato, Hamilton, New Zealand

**Keywords:** Indigenous peoples, Research reporting, Best practice, Equity

## Abstract

**Background:**

Research reporting guidelines are increasingly commonplace and shown to improve the quality of published health research and health outcomes. Despite severe health inequities among Indigenous Peoples and the potential for research to address the causes, there is an extended legacy of health research exploiting Indigenous Peoples. This paper describes the development of the CONSolIDated critERtia for strengthening the reporting of health research involving Indigenous Peoples (CONSIDER) statement.

**Methods:**

A collaborative prioritization process was conducted based on national and international statements and guidelines about Indigenous health research from the following nations (Peoples): Australia (Aboriginal and Torres Strait Islanders), Canada (First Nations Peoples, Métis), Hawaii (Native Hawaiian), New Zealand (Māori), Taiwan (Taiwan Indigenous Tribes), United States of America (First Nations Peoples) and Northern Scandinavian countries (Sami). A review of seven research guidelines was completed, and meta-synthesis was used to construct a reporting guideline checklist for transparent and comprehensive reporting of research involving Indigenous Peoples.

**Results:**

A list of 88 possible checklist items was generated, reconciled, and categorized. Eight research domains and 17 criteria for the reporting of research involving Indigenous Peoples were identified. The research reporting domains were: (i) governance; (ii) relationships; (iii) prioritization; (iv) methodologies; (v) participation; (vi) capacity; (vii) analysis and findings; and (viii) dissemination.

**Conclusions:**

The CONSIDER statement is a collaborative synthesis and prioritization of national and international research statements and guidelines. The CONSIDER statement provides a checklist for the reporting of health research involving Indigenous peoples to strengthen research praxis and advance Indigenous health outcomes.

## Background

Health research is an effective tool to advance wellbeing and improve health outcomes and can be used to identify, monitor, and address health inequities [1–3].

Despite severe health disparities among Indigenous Peoples and the potential of research to identify these, there is an extended legacy of health research exploiting Indigenous Peoples [4, 5]. It has been argued that research conducted “on” Indigenous Peoples have not improved Indigenous health outcomes but perpetuated systemic health inequities and geopolitical dominance by non-Indigenous institutions [6–9]. Failure to utilize research approaches that recognize and account for the ongoing harmful impacts of colonization and that advance Indigenous participation, knowledge, and priorities will continue to impede improvement in Indigenous health outcomes [2, 5, 10]. Hence there is a need for research praxis that critically reflects on the causative factors of inequity to positively impact health outcomes [11, 12].

Research reporting guidelines are increasingly commonplace, and such statements have been shown to improve the quality of published health research and health outcomes [11, 13–17]. This improvement has extended to recommendations on population health and policy, global health research, health estimates, and systematic reviews with a focus on health equity [18–21]. However, there are minimal guidelines for strengthening the reporting of research that explicitly involves Indigenous Peoples to advance Indigenous health, except national statements regarding ethics and funding requirements [22–26]. Researchers have detailed how strengthening research responsiveness is a vital tool in addressing health equity [10, 24, 27].

This paper describes the development of the CONSolIDated critERtia for strengthening the reporting of health research involving Indigenous Peoples (CONSIDER) statement. The CONSIDER statement is a collaborative synthesis and prioritization of existing national and international statements and guidelines.

## Methods

### Search strategy

A search strategy for Indigenous health research and ethics guidelines was undertaken. An initial search using the term “Indigenous” and MeSH terms “ethics, research,” “standards” and exploded MeSH term “guidelines” was conducted. The search strategy was broadened to include the search terms “Aboriginal,” “First Nations” and “Māori.” Information sources were restricted to those involving Indigenous Peoples who continue to experience colonization but insist on decolonized approaches to research, including Indigenous leadership in the development of research and ethics guidelines [28]. The search was performed in Google Scholar and PubMed databases (October 2018). National research and policy websites were searched for national level policy documents.

The eligible research/ethics guidelines from the following nations (Peoples) were included; Australia (Aboriginal and Torres Strait Islanders), Canada (First Nations Peoples, Métis), Hawaii (Native Hawaiian), New Zealand (Māori), Taiwan (Taiwan Indigenous Tribes), United States of America (First Nations Peoples) and Northern Scandinavian countries (Sami).

### Data extraction and synthesis

The CONSIDER statement working group consisted of health researchers, health practitioners, epidemiologists, and methodologists from Australia and Aotearoa New Zealand, all with expertise in Indigenous health and health equity. Three CONSIDER statement working group members (TH, SCP, and SP) independently reviewed the seven eligible guideline reports and extracted the critical criteria for transparent and comprehensive reporting of research involving Indigenous Peoples [22, 25, 26, 29–31]. From these reports, a list of 88 possible checklist items was generated. The three working group members convened to discuss each of the checklist items in turn, and a consensus was reached about whether to retain, merge, or omit each item. The working group members then identified core domains of research conduct and practice under which each checklist item was categorized. The checklist was subsequently reviewed and revised by the entire working group, via an iterative process to ensure that successive changes reflected discussions. All working group members then agreed on the eight domains and 17 checklist items.

## Results

### CONSIDER statement: content and rationale

The CONSIDER statement contains eight research domains and 17 criteria for the reporting of research involving Indigenous Peoples. The CONSIDER statement aims to strengthen research practices and reporting to enhance research conduct and dissemination to support indigenous health equity (Table 1). The checklist includes the research domains of (i) governance; (ii) relationships; (iii) prioritization; (iv) methodologies; (v) participation; (vi) capacity; (vii) analysis and findings; and (viii) dissemination.

**Table 1:** Checklist of items to include when reporting health research involving Indigenous Peoples

Item Checklist Item	
Governance	
1.	Describe partnership agreements between the research institution and Indigenous-governing organization for the research, (e.g., Informal agreements through to MOU (Memorandum of Understanding) or MOA (Memorandum of Agreement)).
2.	Describe accountability and review mechanisms within the partnership agreement that addresses harm minimization.
3.	Specify how the research partnership agreement includes protection of Indigenous intellectual property and knowledge arising from the research, including financial and intellectual benefits generated (e.g., development of traditional medicines for commercial purposes or supporting the Indigenous community to develop commercialization proposals generated from the research).
Prioritization	
4.	Explain how the research aims emerged from priorities identified by either Indigenous stakeholders, governing bodies, funders, non-government organization(s), stakeholders, consumers, and empirical evidence
Relationships (Indigenous stakeholders/participants and Research team)	
5.	Specify measures that adhere and honor Indigenous ethical guidelines, processes, and approvals for all relevant Indigenous stakeholders, recognizing that multiple Indigenous partners may be involved, e.g., Indigenous ethics committee approval, regional/national ethics approval processes.
6.	Report how Indigenous stakeholders were involved in the research processes (i.e., research design, funding, implementation, analysis, dissemination/recruitment).
7.	Describe the expertise of the research team in Indigenous health and research.
Methodologies	
8.	Describe the methodological approach of the research including a rationale of methods used and implication for Indigenous stakeholders, e.g., privacy and confidentiality (individual and collective)
9.	Describe how the research methodology incorporated consideration of the physical, social, economic and cultural environment of the participants and prospective participants. (e.g., impacts of colonization, racism, and social justice). As well as Indigenous worldviews.
Participation	
10.	Specify how individual and collective consent was sought to conduct future analysis on collected samples and data (e.g., additional secondary analyses; third-parties accessing samples (genetic, tissue, blood) for further analyses).
11.	Described how the resource demands (current and future) placed on Indigenous participants and communities involved in the research were identified and agreed upon including any resourcing for participation, knowledge, and expertise
12.	Specify how biological tissue and other samples including data were stored, explaining the processes of removal from traditional lands, if done, and of disposal.
Capacity	
13.	Explain how the research supported the development and maintenance of Indigenous research capacity (e.g., specific funding of Indigenous researchers).
14.	Discuss how the research team undertook professional development opportunities to develop the capacity to partner with Indigenous stakeholders?
Analysis and interpretation	
15.	Specify how the research analysis and reporting supported critical inquiry and a strength-based approach that was inclusive of Indigenous values.
Dissemination	
16.	Describe the dissemination of the research findings to relevant Indigenous governing bodies and peoples.
17.	Discuss the process for knowledge translation and implementation to support Indigenous advancement (e.g., research capacity, policy, investment).

The scope of the CONSIDER statement is all forms of original health research, regardless of methodologies, that includes a substantial Indigenous component including research: conducted on Indigenous lands; in which Indigenous identity is a criterion for participation; that seeks Indigenous knowledge; in which identity or membership of an Indigenous community is used as a variable for data analysis in which interpretation of data refers directly to Indigenous Peoples; or research that is likely to affect the health of Indigenous Peoples. CONSIDER is designed to enhance research practices with and involving Indigenous Peoples. It is anticipated that to strengthen research reporting, investigators should report whether or not each CONSIDER checklist item has been addressed during research design or conduct. The CONSIDER statement is not intended to reproduce general ethical guidelines.

To elaborate on the CONSIDER statement, we have produced supporting explanations of each domain and checklist item. The CONSIDER working group approved additional revisions before finalization of the statement.

### The CONSIDER checklist

#### Domain 1: research governance

Research governance emphasizes reporting of the relationship building that occurred between the research institution hosting the research and Indigenous organizations with oversight responsibilities to the participants and communities involved in the research. Governance relates to partnerships between the research institution(s) and Indigenous organization(s) to recognize the centrality of Indigenous self-determination and leadership in research conduct and to provide an accountability mechanism by which the host research institution aims to meet the principles, expectations, priorities, and values of Indigenous research stakeholder(s). Reporting about research governance includes an understanding that acknowledges partnerships can change in nature, scope, or goals over time. Therefore it is helpful for research institutions to plan regular reviews of any partnership agreement to sustain equity in the partnership over time. Research governance relationships should include reporting of any a priori process to ensure that the proposed research adheres to the principles of ethical conduct and addresses and minimizes the potential for harm to Indigenous Peoples [32, 33]. To achieve this, researchers must report on the organizational structures that safeguard ethical research partnerships with Indigenous organizations. These might be operationalized as Memoranda of Understanding (MOU) and Memoranda of Agreement (MOA). Institutional agreements should be written to avoid research praxis as a further expression of colonization and appropriation and aim to foster a partnership that maximizes the benefits of research to Indigenous health advancement.

1. *Partnership agreement between the research institution and Indigenous governing organization or collective (*e.g.*, MOU or MOA):* Reporting of the critical elements of a partnership agreement between the research institute and the Indigenous governance structures enables transparency and sets expectations for research conduct and specifically research and data ownership, custodianship, access, and permission. A partnership agreement between a research institution and the Indigenous stakeholder(s) can be symbolic, however the operational component of the partnership will articulate a shared understanding of the resourcing, priority, intent, scope, conduct, knowledge sharing, intellectual property gains, and dissemination of the research to benefit Indigenous health outcomes and development.
2. *Accountability mechanism to address harm minimization:* Research practices can lead directly to harm to Indigenous stakeholders, for example, the repeated use of tissue samples and data without specific permission during the consent process that violates Indigenous knowledge and custom [4]. To strengthen the accountability of the research, the research institution should have appropriate procedures and protocols to avoid harm and hold researchers accountable during both research conduct and dissemination.
3. *Protection of Indigenous intellectual property and knowledge*: Research with Indigenous stakeholders should recognize and protect Indigenous knowledge contributing to and arising from the research. This principle is embodied by Article 31 of the United Nations Declaration on the Rights of Indigenous Peoples that Indigenous Peoples “have the right to maintain, control, protect, and develop their cultural heritage, traditional knowledge and traditional cultural expressions, as well as the manifestations of their sciences, technologies, and cultures, including human and genetic resources, seeds, medicines, knowledge of the properties of fauna and flora, oral traditions, literatures, designs, sports and traditional games, and visual and performing arts. They also have the right to maintain, control, protect, and develop their intellectual property over such cultural heritage, traditional knowledge, and traditional cultural expressions” [32]. Research groups should clarify how their governing institute preserved and enacted Indigenous control, protection, and development of intellectual property, including economic and intellectual benefits arising from participation in specific research.

#### Domain 2: research prioritization

Researchers and research groups are recommended to explain how the research was prioritized and whether the prioritization process involved Indigenous stakeholders, empirical evidence, governing bodies, and funding agencies. The priorities of the Indigenous stakeholders, government/funding organizations, and research group(s), may differ, and a statement about how any differences were considered and reconciled would strengthen the research reporting.

4) *How the research aims emerged from research priorities:* Explanation of whether Indigenous stakeholders (including individuals and communities) participated in the identification of research aims demonstrates how researchers/research groups perceived their role in research to advance Indigenous health. A description of the process of developing research aims from any reported research priorities can also help with the assessment of whether the research objectives were likely to represent Indigenous stakeholders’ health priorities. Analysis of prior research can provide insights into methodological approaches previously employed, are consistent with findings across community and clinical settings, and whether the research has been completed already. If a systematic analysis is not available, the researchers should report any attempt to explore and communicate existing evidence during the research planning and consultation phases.

#### Domain 3: research relationships

This domain refers to the relationships and processes undertaken by the researcher or research group with the Indigenous partners in the research process (individually and collectively).

5) *Adherence and honoring Indigenous ethical guidelines, processes and approvals:* Research groups should assure users of the research that the research conduct met ethical guidelines and observed human rights, including meaningful engagement and reciprocity between the researcher and the individuals or stakeholders involved in the study [22, 24, 29, 32, 34]. Researchers should describe the ethical processes relevant to Indigenous stakeholders that were explicitly undertaken. This may include processes involving multiple regulatory and ethics bodies. Researchers should report how any potential conflicts in the requirements between different regulatory agencies were understood and reconciled or addressed. Informative descriptions of ethical processes can increase the accountability of researchers to specific conditions (state, territory, nation, or local) and should provide information about ongoing consultation and monitoring of the research process [35].
6) *Involvement of Indigenous stakeholders in the research processes*: Researchers should specify how Indigenous participants and stakeholders were involved in research processes to assist in understanding how the research enacted principles of self-determination. A lack of involvement and partnership with Indigenous research participants results in minimal improvement of health outcomes and Indigenous development, reinforcing non-Indigenous priorities and structures [5, 36, 37]. Empirical evidence suggests that effective implementation of research is supported by involving Indigenous health workers and community control organizations and partnerships [38]. Generally, it is appropriate to match the expertise and capacity of participants with involvement in the research process or provide training and support to enable effective partnership. Consideration of specific resourcing for Indigenous partnerships is appropriate and should be reported by researchers.
7) *The expertise of the research team in Indigenous health research*: Researchers should describe the expertise of the research team in the conduct of research involving Indigenous Peoples. Specific expertise may encompass partnership capacity, knowledge of the impacts of colonization and racism on Indigenous health outcomes, participatory research skills, policy and funding relationships, methodological experience, and ethical and intellectual property knowledge. The credibility of the research process and outputs can be increased by the understanding that the researcher or research group has specific expertise in research involving Indigenous participants. Research groups should seek particular expertise during research inception to ensure the range of capacities required for research conduct is adequately represented during the time course of the work.

#### Domain 4: research methodologies and methods

Research methodologies include techniques that articulate “the context in which research questions are conceptualized and designed” and consider “the implications of research for its participants and their communities.” [5] Indigenous methodologies consider the “institution of research, its claims, its values and practices, and its relationships to power.” [5] Research evaluating Indigenous health incorporates considerations of power, and broader political and social structures can support and explore an understanding of influences on Indigenous health including colonization, racism and social injustice. Likewise, methodologies should inform research methods, and therefore should reflect research methods that are aligned with the social realities of the Indigenous stakeholders.

8) *Methodological approach:* A description of the methodological approach should identify the theoretical framework that underpins the study. The inclusion of the methodological approach highlights how the researchers considered and contextualized their research aims, analyses, and findings. Including Indigenous quantitative and qualitative methods that have known positive impacts on Indigenous stakeholders [10].
9) *Consideration of physical, social, economic, and cultural environment of Indigenous stakeholders including implications of colonization, racism, and social injustice*: Indigeneity is a marker of exposure to risk factors that contribute to inequitable distribution of power, money, and resources [39]. Examination of these risk factors is considered necessary to improve health. A description of how researchers examined the political and social context of their research should be provided to clarify how the social, political, and economic environment informed analysis and interpretation of findings. Researchers should seek to avoid deficit assumptions and language, and specifically, avoid placing the locus of responsibility for inequities on Indigenous communities. Ensuring that critiques of colonial and racist systems are accounted for within the design and implementation of the research.

#### Domain 5: research participation

Key considerations about Indigenous participation in the research should include; ethical considerations of the data gathered, including data confidentiality, linkage or sharing, the burden of research participation on Indigenous communities, storage, and removal of biological specimens, and future use of Indigenous data and knowledge.

10) *Individual and collective consent to conduct future analyses on collected samples and data:* Inappropriate secondary use of Indigenous data and biological samples including DNA samples without consent has occurred throughout history. This is directly counter to the principles of data sovereignty and self-determination [40]. Secondary use requires further ethics review and approval. Research teams should describe any new consent processes that occurred for re-analysis or secondary use of collected samples. Any transfer of samples to a third party, not included in the primary consent process, then that party should specify the further consent process that occurred for data and sample transfer and secondary use, or state that this did not occur.
11) Resource *demands (present/ future/cultural/emotional/intellectual) placed on Indigenous participants and participant communities:* Recognizing and addressing the burden of research participation on Indigenous participants and communities is important as an expression of reciprocity and good research practice. Considering the resource demands of the research on Indigenous participants is particularly relevant when there has not been full consultation and partnership to align the research with Indigenous priorities, or when the research could be carried out elsewhere or using different methods [41]. Researchers should consider how the participation of Indigenous investigators and participants in the research is resourced, including providing adequate time and funding for face-to-face consultation, employment of local Indigenous Peoples in the research conduct, and remuneration for the work and expertise of Indigenous advisory or reference groups. Research groups should specify how they avoided placing pressure on local communities to accept externally funded or national projects and describe the negotiation process or agreement that was reached on study resourcing for Indigenous participation through the life course of the research.
12) *Storage and removal of biological tissues and other samples and data from traditional lands (if done), and disposal:* When entering into a research agreement, there should be a frank discussion about how data and samples can and should be used, and what can and cannot be done with the samples. Indigenous Peoples expectations may include the return samples and tissues after completion of the research. Some research institutions have policies that research samples become the property of that institution and Indigenous organizations may have requirements for control of data and samples, including a return of samples to the participant or organizations. Researchers should specify any negotiation between the research team and the Indigenous organization about the control, storage, and disposal of data. The research team should recognize the proprietary interests of Indigenous Peoples in data and biological samples and sample removal from traditional lands or disposal of data and samples should only occur by agreement. This includes individual and collective consent before sample and data collection. Research teams should specify the sample and data management plan that was detailed in the research agreement within any research outputs. Indigenous data sovereignty demands that research practices must be transparent about how data is stored, governed, and used, including whether individual information will be removed from traditional lands, e.g., transferred to international databases or tissue banks [23, 36].

#### Domain 6: research capacity

The reporting of this criterion ensures that researchers, research groups and research institutions recognize and acknowledge the rights of Indigenous Peoples to have self-determination in the achievement of research. This self-determination includes ownership and control of research through the support and resourcing of Indigenous research capacity. This also includes the professional development of research groups to increase their research skill set when working in partnership with Indigenous stakeholders.

13) *Research teams supporting the development and maintenance of Indigenous research capacities:* Research should be of benefit to Indigenous stakeholders as well as to the research group. Reporting the capacity-building components of the research process for Indigenous health research provides an understanding that the research team values this aspect of working with Indigenous stakeholders and recognizes the potential impact and benefits of research with communities. Increasing Indigenous health research capacity should be by mutual negotiation to maximize the relevance and sustainability of any program or activity. A research team may specify research education and training as part of the research protocol or agreement.
14) *Professional development by the research team to develop a capacity to partner with Indigenous Peoples:* Research teams must conduct research with Indigenous Peoples that recognizes Indigenous values and worldview, and that meets the expectations of observing protocols and customs. Research teams should report any professional development training that strengthens their work with Indigenous partners during the entire research process, e.g., language/cultural understanding. This will add credibility that the research team prioritizes respect and cultural responsiveness during research work.

#### Domain 7: research analysis and interpretation

Research groups must report on the inclusion of critical inquiry and strength-based approaches to the research analysis, including the incorporation/valuing of cultural beliefs or values into the research findings, including the involvement of Indigenous stakeholders in the analysis and interpretation of the research.

15) *Analysis and reporting supported critical inquiry and strength-based approach*: The tenet of a strength-based approach is ensuring that research does not perpetuate or reaffirm stereotypical beliefs. The inclusion of Indigenous stakeholders in the analysis, interpretation, and reporting of the research may assist in reducing the risks of research interpretations and outputs that advance a theory or knowledge conceptualization based on non-evidence based understanding of Indigenous health [42]. This also includes how Indigenous stakeholders have been acknowledged in the research, including principles of equity within authorship that align with partnership research approaches and contributions of Indigenous knowledge and expertise.

#### Domain 8: research dissemination

The final criteria specify how research teams disseminate the research outcomes to the appropriate Indigenous stakeholders in parallel with standard pathways for research dissemination and knowledge translation. It is widely understood that “dissemination of research is essential to achieve social value.” [33] Therefore, the social value of disseminating research outcomes to Indigenous stakeholders is an effective strategy in knowledge translation and partnership [42]. This enables Indigenous stakeholders to hold researchers accountable for their research praxis within their communities as well as utilizing the information to monitor organizations and to advocate for policy change and resources.

16) *Dissemination of research outputs*: Indigenous health research is relevant to Indigenous communities and organizations, policy makers, Indigenous and non-Indigenous health service providers, and clinicians as well as other research teams and the wider public. The methods to exchange information about the research should be tailored to the user. The research agreement is the optimal mechanism to establish the research dissemination and translation process with peak stakeholders. The exchange of research findings with Indigenous stakeholders and relevant health service and policy-makers should be outlined. Including whether this process was negotiated with Indigenous stakeholders, leaders, and organizations.
17) *Process for knowledge translation to support Indigenous health advancement.* The aims of research involving Indigenous stakeholders include the improvement of wellbeing and health services and addressing inequity and injustice. To this end, research groups should describe how their research plan and agreement was communicated, translated, and implemented aligned with these goals. This includes accessible, ongoing, and reciprocal communication with the community, health sector, non-profit, and governmental organizations including those under Indigenous control. Any use of cultural knowledge, traditions, and practices arising should be by permission and agreement.

## Discussion

The CONSIDER reporting criteria were developed from a conceptual synthesis of ethics and research guidelines for research involving Indigenous Peoples from Australia (Aboriginal and Torres Strait Islanders), Canada (First Nations Peoples, Métis), Hawaii (Native Hawaiian), New Zealand (Māori), Taiwan (Taiwan Indigenous Tribes), United States of America (First Nations Peoples), and Northern Scandinavian countries (Sami). The criteria provide a checklist for the reporting of equitable research practices. The checklist items are focused on the reporting of Indigenous participation in research including, who is leading the investigation, participant recruitment, participant confidentiality, and the consenting process for future analysis of tissue samples and database information. These practices not only include a partnership with Indigenous stakeholders but also research that is Indigenous-led, controlled and financed by Indigenous interests, whereby the non-Indigenous population becomes a partner.

There is minimal evidence that these criteria are being widely implemented in research praxis. The criteria provide the opportunity for researchers, research governing institutions and research funders to ensure an accountable ‘closing of the research loop,’ to increase research accountability [5, 43]. Greater adherence to the criteria will strengthen the research process and have a positive impact on research relationships with Indigenous Peoples.

## Conclusion

The CONSIDER statement provides a checklist to strengthen the reporting of Indigenous health research. The statement is a collaborative synthesis of publicly available guidelines for ethical research conduct involving Indigenous Peoples in nations in which ongoing colonization is present (Australia, Canada, New Zealand, Hawaii, Taiwan, United States of America, and Northern Scandinavia). The CONSIDER statement provides criteria for reporting of research aimed to strengthen Indigenous health research and to advance Indigenous health outcomes and development.

## Data Availability

All ethics and research guidelines used for this article are publically available.

## Abbreviations

CONSIDER: Consolidated Criteria for strengthening the reporting of health research involving Indigenous Peoples
MOA: Memorandum of Agreement
MOU: Memorandum of Understanding
